# Silencing of augmenter of liver regeneration inhibited cell proliferation and triggered apoptosis in U266 human multiple myeloma cells

**DOI:** 10.1590/1414-431X20176139

**Published:** 2017-08-31

**Authors:** H.Q. Zeng, Y. Luo, S.F. Lou, Q. Liu, L. Zhang, J.C. Deng

**Affiliations:** 1 Department of Hematology, the Second Affiliated Hospital of Chongqing Medical University, Chongqing, China; 2 Institute of Viral Hepatitis, the Second Affiliated Hospital of Chongqing Medical University, Chongqing, China; 3 Department of Nephrology, the Second Affiliated Hospital of Chongqing Medical University, Chongqing, China

**Keywords:** Apoptosis, Augmenter of liver regeneration, Hematologic cancer, Proliferation, Small interfering RNA

## Abstract

Augmenter of liver regeneration (ALR) is a thermostable cytokine that was originally identified to promote the growth of hepatocytes. This study was conducted to explore the expression and function of ALR in multiple myeloma (MM), a common hematologic malignancy. Real-time PCR and western blot analysis were performed to detect the expression of ALR in U266 human MM cells and healthy peripheral blood mononuclear cells (PBMCs). U266 MM cells were exposed to 20 or 40 μg/mL of recombinant ALR and tested for cell proliferation. Small interfering RNA-mediated silencing of ALR was done to investigate the role of ALR in cell proliferation, apoptosis, and cytokine production. Compared to PBMCs, U266 MM cells exhibited significantly higher levels of ALR at both the mRNA and protein levels. The addition of recombinant ALR protein significantly promoted the proliferation of U266 cells. In contrast, knockdown of ALR led to a significant decline in the viability and proliferation of U266 cells. Annexin-V/PI staining analysis demonstrated that ALR downregulation increased apoptosis in U266 MM cells, compared to control cells (20.1±1.1 *vs* 9.1±0.3%, P<0.05). Moreover, ALR depletion reduced the Bcl-2 mRNA level by 40% and raised the Bax mRNA level by 2-fold. Additionally, conditioned medium from ALR-depleted U266 cells had significantly lower concentrations of interleukin-6 than control cells (P<0.05). Taken together, ALR contributed to the proliferation and survival of U266 MM cells, and targeting ALR may have therapeutic potential in the treatment of MM.

## Introduction

Multiple myeloma (MM) is a malignant tumor originating from plasma cells, accounting for over 10% of all hematologic cancers (1). In the past decades, many novel agents have been developed to treat MM, improving the 5-year survival rate to about 50% (2). However, MM is still incurable and has a high incidence of drug resistance and relapse (3). A number of cytokines in bone marrow niche have been identified to be implicated in MM development and progression (4). For instance, interleukin (IL)-6 is required for the growth of MM cells (5). Silencing of IL-6 in mesenchymal stromal cells has been reported to interfere with MM cell growth (6). IL-10, tumor necrosis factor-alpha (TNF-α), and vascular endothelial growth factor (VEGF) also affect the biology of MM cells (4,7).

Augmenter of liver regeneration (ALR) is a thermostable cytokine that was originally identified to promote the growth of hepatocytes (8). Recent studies indicate that ALR is dysregulated in multiple cancers and plays an important role in tumor growth and progression (9–11). For instance, ALR expression is elevated and correlates with aggressive parameters in colon cancer tissues (9). Downregulation of ALR was reported to induce apoptosis in human glioma cells (11). In Jurkat T leukemia cells, ALR was found to attenuate apoptotic response and reduce the sensitivity to vincristine (12). However, the biological roles of ALR in MM cells are still unclear.

Therefore, in this study, we measured the expression of ALR in MM cells and investigated its role in cell proliferation, apoptosis, and cytokine production.

## Material and Methods

### Cell culture and treatment

Human MM cell line U266 was obtained from the Institute of Viral Hepatitis of Chongqing University of Medical Sciences (Chongqing, China). Human peripheral blood mononuclear cells (PBMCs) were kindly provided by Department of Hematology, the Second Hospital Affiliated to Chongqing University of Medical Sciences. Cells were cultured in RPMI-1640 medium containing 10% fetal bovine serum (FBS), 100 U/mL penicillin and 100 μg/mL streptomycin; all from Invitrogen (USA). Recombinant human ALR protein was obtained as described previously (11). U266 MM cells at ∼80% confluence were exposed to 20 or 40 μg/mL of recombinant ALR for indicated times and tested for proliferation. Untreated cells were used as control.

### Quantitative real-time PCR (qRT-PCR) analysis

Total RNA from U266 cells was extracted with TRIzol reagent (Invitrogen) and reverse transcribed to cDNA. Real-time PCR was performed using the PrimeScript RT-PCR Reagent Kit with SYBR Green following the manufacturer's instructions (TaKaRa, China). PCR primers are summarized in Table 1. The relative mRNA levels were determined by normalizing to that of β-actin mRNA using the comparative cycle threshold method (13).

**Table 1.** PCR primers used in this study.

Gene	Sequence (5′-3′)
hALR	Forward: AAGGTGAGGCTGGGAATTT
	Reverse: GTCTTCATGTCGCGCTTCT
Bcl-2	Forward: GCCCTGTGGATGACTGAGTA
	Reverse: CAGCCAGGAGAAATCAAACA
P53	Forward: AGACCCAGGTCCAGATGAAG
	Reverse: TTTCTGGGAAGGGACAGAAG
Bax	Forward: GACTCCTCAAGCCTCCTCAC
	Reverse: GAGAGGGCACCACTGTGAC
P21	Forward: CTTCGACTTTGTCACCGAGA
	Reverse: CGTGGGAAGGTAGAGCTTG
Survivin	Forward: AGGTGAGAAGTGAGGGAGGA
	Reverse: CACATTCACTGTGGAAGGCT

### Western blot analysis

Cells were lysed in RIPA buffer (BioTeke Corporation, China) containing protease inhibitors (Roche Applied Science, USA). Protein samples were separated by sodium dodecyl sulfate (SDS)-polyacrylamide gel electrophoresis and transferred onto a nitrocellulose membrane. The membrane was incubated with mouse anti-human ALR monoclonal antibody (1:200 dilution; sc-365885, Santa Cruz Biotechnology, USA) or mouse anti-human β-actin monoclonal antibody (1:2000 dilution; sc-130300, Santa Cruz Biotechnology) at 4°C overnight. After washing, the membrane was incubated with horseradish peroxidase-conjugated anti-mouse IgG for 1 h. Protein signals were developed by enhanced chemiluminescence (Amersham Biosciences, USA) and quantified using the Quantity One software (Bio-Rad Laboratories, USA).

### Transfection with ALR shRNA

Small hairpin RNA (shRNA) targeting human ALR mRNA and negative control shRNA were purchased from Genechem (China). U266 cells were transfected with control or ALR shRNA using Lipofectamine 2000 (Invitrogen) according to the manufacturer's instructions. Transfection efficiency (80%) was achieved, which was determined by co-transfection of a fluorescently labeled non-targeting small interfering RNA (Genechem).

### Cell proliferation assay

Cells were plated in 96-well plates at a density of 1×10^4^ cells per well. After 24-, 48-, and 72-h incubation, cells were collected. The cell number was determined by the 3-(4,5-dimethylthiazol-2-yl)-2,5-diphenyl-2H-tetrazolium bromide (MTT) assay (BioTeke Corporation). Absorbance was recorded at 570 nm using a microplate reader.

### BrdU incorporation assay

Cell proliferation was measured using the 5-bromo-2′-deoxy-uridine (BrdU) Cell Proliferation Assay Kit (Roche Applied Science) according to the manufacturer's instructions. BrdU incorporation was determined spectrophotometrically by measuring absorbance at 450 nm.

### Apoptosis analysis by flow cytometry

Apoptosis was measured using the Annexin-V FITC Apoptosis Detection Kit (Roche Applied Science). In brief, cells were fixed in 70% ethanol and resuspended in staining solutions containing fluorescein isothiocyanate (FITC)-conjugated Annexin-V and propidium iodide (PI). Cells were analyzed by a flow cytometer (BD FACSCanto II, Becton Dickinson, USA).

### Preparation of conditioned medium

Conditioned medium was prepared as described previously (14). In brief, U266 cells transfected with control or ALR shRNA were cultured in fresh medium containing 10% FBS for 72 h. Cells were then incubated in serum-free medium for another 24 h. The conditioned medium of cells was collected and centrifuged at 2000 *g* for 5 min at 4°C. The supernatant was filtered through a 0.22-µm membrane and stored at -20°C until use.

### Measurement of cytokine levels by enzyme-linked immunosorbent assay (ELISA)

For measurement of IL-6, IL-10, TNF-α, and VEGF levels in conditioned medium, commercially available ELISA kits (BioTeke Corporation) were used, according to the manufacturer's instructions.

### Statistical analysis

Data are reported as means±SD and were analyzed by Student's *t*-test or one-way ANOVA with the Tukey test. Statistically significant differences were defined as P<0.05.

## Results

### ALR expression was elevated in U266 MM cells

**Figure 1.**
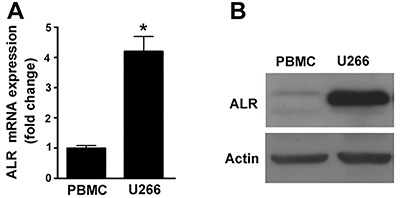
Augmenter of liver regeneration (ALR) expression was elevated in U266 MM cells. *A*, qRT-PCR, and *B*, western blot analysis of the mRNA and protein levels of ALR, respectively, in U266 multiple myeloma cells and healthy peripheral blood mononuclear cells (PBMCs). Representative blots from 3 independent experiments are shown in *B*. *P<0.05 *vs* PBMC; n=3 (Student *t*-test).

As determined by qRT-PCR, the mRNA level of ALR was 4-fold higher in U266 MM cells than that in PBMCs from healthy individuals (P<0.05; Figure 1A). Western blot analysis confirmed that ALR protein was abundantly expressed in U266 MM cells, but at low levels in healthy PBMCs (Figure 1B).

### ALR stimulated the proliferation of U266 MM cells

**Figure 2.**
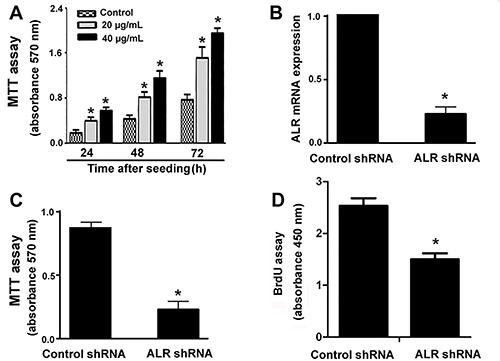
Augmenter of liver regeneration (ALR) regulated the proliferation of U266 multiple myeloma (MM) cells. *A*, U266 MM cells were exposed to 20 or 40 μg/mL recombinant ALR protein for 24-72 h and cell viability was examined by the MTT assay. ALR treatment significantly promoted the proliferation of U266 MM cells. *P<0.05 *vs* untreated control cells; n=6 (one-way ANOVA with the Tukey test). *B*, qRT-PCR analysis of the ALR mRNA level in U266 cells transfected with control or ALR shRNA; n=3. *C*, MTT assay was done to measure the viability of U266 cells transfected with control or ALR shRNA after culturing for 72 h; n=6. *D*, Cell proliferation was determined by BrdU incorporation assay after culturing for 72 h. ALR silencing significantly suppressed the proliferation of U266 MM cells. *P<0.05 *vs* the control shRNA group; n=6 (Student *t*-test).

U266 MM cells were exposed to 20 or 40 μg/mL recombinant ALR protein for 24-72 h and cell proliferation was examined. Compared to control cells, ALR-treated U266 MM cells had significantly higher proliferation (P<0.05; Figure 2A). Moreover, ALR at higher concentrations yielded more profound growth promotion. To complement the overexpression experiments, shRNA-mediated targeting of ALR was conducted. qRT-PCR analysis revealed that delivery of ALR-targeting shRNA led to a significant inhibition of ALR expression in U266 MM cells, compared to control shRNA-transfected cells (P<0.05; Figure 2B). It was found that the viability was significantly lower in ALR-depleted U266 MM cells (Figure 2C). Consistently, BrdU incorporation assay showed that there was a 47% reduction in proliferation in U266 MM cells transfected with ALR shRNA after 72-h culture, compared to control cells (P<0.05; Figure 2D).

### ALR silencing induced apoptosis in U266 MM cells

**Figure 3.**
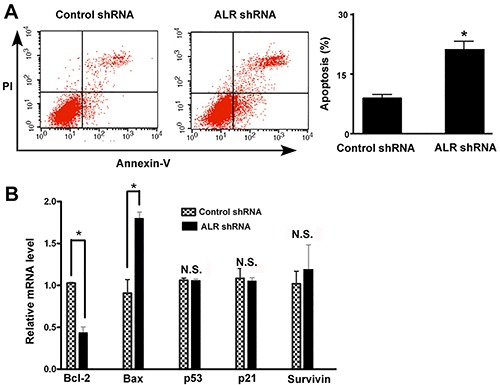
Augmenter of liver regeneration (ALR) silencing induced apoptosis in U266 multiple myeloma cells. *A*, Flow cytometric analysis of apoptosis in U266 cells transfected with control or ALR shRNA after Annexin-V/PI staining. *B*, qRT-PCR analysis of indicated transcripts in U266 cells transfected with control or ALR. *P<0.05 *vs* the control shRNA group; n=3. N.S. indicates no significance between the 2 groups (Student *t*-test).

Annexin-V/PI staining analysis showed that ALR knockdown significantly triggered apoptosis in U266 MM cells, compared to control cells (20.1±1.1 *vs* 9.1±0.3%, P<0.05; Figure 3A). Several key apoptosis-related genes were also measured by qRT-PCR analysis (Figure 3B). The results demonstrated that ALR downregulation led to 40% decline the Bcl-2 mRNA level and a 2-fold elevation in the Bax mRNA level, relative to control cells. However, p53, p21, and Survivin mRNA levels remained unchanged.

### ALR depletion impaired the production of IL-6 in U266 MM cells

**Figure 4.**
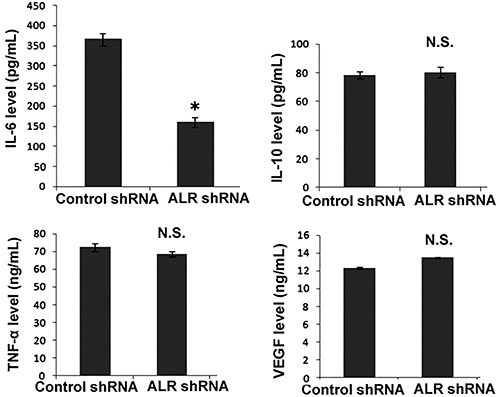
Augmenter of liver regeneration (ALR) depletion reduced the production of IL-6 in U266 multiple myeloma cells. Measurement of IL-6, IL-10, TNF-α, and VEGF levels in conditioned medium from transfected U266 cells by ELISA. *P<0.05 *vs* the control shRNA group; n=3. N.S. indicates no significance between the 2 groups (Student *t*-test).

Finally, we examined the effect of ALR silencing on the production of key cytokines involved in cell proliferation and survival. The results of ELISA indicated that the IL-6 concentration in conditioned medium from ALR-depleted U266 MM cells was significantly lower than that from control cells (P<0.05; Figure 4). However, no significant difference in the production of IL-10, TNF-α, and VEGF was noted between control and ALR-depleted U266 MM cells (P>0.05).

## Discussion

ALR is dysregulated in a number of solid tumors such as hepatocellular carcinoma, cholangiocellular carcinoma (15), and colorectal cancer (9). The present study extended this finding to MM cells and showed that ALR was significantly upregulated in U266 MM cells relative to healthy PBMCs. We found that both ALR mRNA and protein levels were consistently increased in U266 MM cells, suggesting the induction of ALR expression at the transcription level. ALR is well-defined as a hepatic mitogen that facilitates hepatocyte proliferation after liver damage (16). In malignant cells, ALR also displays the ability to modulate cell behaviors (11,12). For instance, it was reported that downregulation of ALR significantly inhibited the growth of hepatocellular carcinoma cells *in vitro* and in xenograft tumors *in vivo* (17). Another study showed that ALR can protect SH-SY5Y human neuroblastoma cells from hydrogen peroxide-induced cell viability loss and apoptosis (18). ALR also confers protection against vincristine-induced cell death in Jurkat T leukemia cells (12). These studies encouraged us to explore the biological roles of ALR in MM cells. Of note, the addition of recombinant ALR to the culture of U266 MM cells led to a significant increase in proliferation. In contrast, silencing of ALR caused a reduction in the proliferation of U266 MM cells. These observations indicate that ALR functioned as a positive regulator of MM growth, which provided a biological explanation for its upregulation in MM.

Induction of apoptosis is an important anticancer therapeutic strategy. Having identified the growth-suppressive activity of ALR downregulation, we next tested the effect of silencing of ALR on the apoptosis of U266 MM cells. As expected, we found that ALR-depleted U266 MM cells displayed significant apoptotic death relative to control shRNA-transfected cells. At the molecular level, the anti-apoptotic Bcl-2 gene was downregulated and the pro-apoptotic gene Bax was upregulated in ALR-depleted U266 MM cells. It has been documented that downregulation of ALR impairs mitochondrial function and induces oxidative damage in T98G glioma cells (11). The Bcl-2 family members play a critical role in mitochondria-dependent apoptosis (19). In response to apoptotic stimuli, the Bax protein can translocate to the mitochondria where it interacts with other Bcl-2 family members, causing cytochrome c release and activation of apoptotic cascade. On the contrary, Bcl-2 shows the ability to block the release of mitochondrial cytochrome c. It is thus suggested that ALR downregulation-elicited apoptosis in MM cells is, at least partially, ascribed to the deregulation of Bax and Bcl-2.

In addition, we also noted that ALR silencing significantly reduced the production of IL-6 in U266 MM cells, compared to control shRNA-transfected cells. The growth of MM cells is coordinated by many cytokines including IL-6 (20). It has been reported that induction of autocrine production of IL-6 is responsible for interferon alpha-induced growth in MM cells (21). IL-6 was reported to attenuate apoptotic response in MM cells after inducing by anti-fas antibodies and dexamethasone (22). Therefore, it is possible that ALR may exert its effects on MM cell proliferation and apoptosis by stimulating IL-6 production. However, the exact mechanism by which ALR modulates IL-6 expression is not yet known.

However, a major limitation of this study is that the biological activity of ALR was evaluated in only one MM cell line. Additionally, the *in vivo* function of ALR needs to be further validated.

In conclusion, our data demonstrated that ALR was overexpressed in U266 MM cells and that silencing of ALR inhibited cell proliferation and triggered apoptosis. The biological activity of ALR in U266 MM cells is causally linked to deregulation of Bcl-2 and Bax and promotion of IL-6 production. These results provide a rationale to investigate the anticancer potential of targeting ALR in animal models of MM.
